# Cultural transmission of traditional songs in the Ryukyu Archipelago

**DOI:** 10.1371/journal.pone.0270354

**Published:** 2022-06-24

**Authors:** Yuri Nishikawa, Yasuo Ihara

**Affiliations:** Department of Biological Sciences, The University of Tokyo, Bunkyoku, Tokyo, Japan; Leiden University, GERMANY

## Abstract

Geographic patterns of cultural variations are affected by how cultural traits are transmitted within and between populations. It has been argued that cultural traits are transmitted in different manners depending on their characteristics; for example, words for basic concepts are less liable to horizontal transmission between populations (i.e., borrowing) than other words. Here we examine the geographic variation of traditional songs in the Ryukyu Archipelago, southwestern islands of Japan, to explore cultural evolution of music with a focus on different social contexts in which songs are sung. Published scores of 1,342 traditional songs are coded using the CantoCore song classification scheme and distances between the songs are calculated from the codings. Neighbor-Net graphs of regions/islands are generated on the basis of the musical distances, and delta scores are obtained to examine the treelikeness of the networks. We also perform analysis of molecular variance (AMOVA) to evaluate the extent of musical diversification among regions/islands. Our results suggest that horizontal transmission between populations has played a greater role in the formation of musical diversity than that of linguistic diversity in the Ryukyu Archipelago and that the social context in which songs are sung has an effect on how they are transmitted within and between populations. In addition, we compare the observed patterns of song diversity among regions/islands with those of lexical and mitochondrial-DNA (mtDNA) diversity, showing that the variation of songs sung in the "work" context are associated with the linguistic variation, whereas no association is found between the musical and genetic variation.

## Introduction

The study of cultural evolution, with the effective use of theories and concepts developed in evolutionary biology, has explored evolutionary dynamics in cultural change driven by transmission and innovation [1–3]. Like horizontal gene transfer in bacteria and archaea [4], the transmission of cultural traits occurs not only from parents to offspring (i.e., vertical transmission; [1]), but also through other pathways such as peer to peer (i.e., horizontal transmission) and from adults to unrelated young (i.e., oblique transmission). In cultural macroevolutionary or phylogenetic studies [5, 6], where populations rather than individuals are taken as a unit of analysis, vertical transmission implies population divergence, in which a daughter population inherits the cultural traits of the parent population, while horizontal transmission occurs between unrelated or related populations through migration and/or cultural exchange.

The relative importance of vertical and horizontal transmission in observed patterns of population-level cultural diversity has long been a matter of debate, and various attempts have been made to disentangle them (e.g., [7–14]). In fact, the relative importance may vary across cultural traits. As a prime example, words for basic concepts, as those on Swadesh’s list of basic vocabulary [15], are considered more resistant to borrowing than other words are and predominantly vertically transmitted. For non-linguistic cultural traits, association with language is often considered as indicative of vertical transmission. Guglielmino et al. [7], for example, examined the associations of cultural traits with language, natural environment, and geography, each capturing the effect of vertical transmission, local adaptation, and horizontal transmission, respectively, in African societies documented in *Ethnographic Atlas* [16]. They suggested that of the six domains of cultural traits investigated ("family and kinship," "economy," "social stratification," "labor division by sex," "house," and "various others"), vertical transmission played a particularly important role in the "family and kinship" domain. In a more recent study to quantify the roles of shared ancestry and geography in the formation of cultural diversity documented in the Western North American Indian database (WNAI; [17]), Towner et al. [10] concluded that both vertical and horizontal transmission are important irrespective of cultural domains.

Music, a supposed human universal [18], is also culturally transmitted both within and between populations. Cultural evolution of music is currently attracting renewed interests [19], beyond earlier attempts as represented by Alan Lomax’s Cantometrics Project [20], by incorporating methods of evolutionary biology. Most relevant to the present context, recent studies have applied methods for the analysis of population-level genetic diversity to analyze musical data across human populations [21–25]. By applying the methods of genetic studies, cultural data can be analyzed quantitatively, and human population history can be revealed from multiple perspectives using both genetic and cultural data. For example, Rzeszutek et al. [22] quantified pairwise distances among 421 traditional songs from 16 Austronesian-speaking populations in Taiwan and the Philippines using the CantoCore music classification scheme [26], and applied the analysis of molecular variance (AMOVA; [27]) to examine the within- and between-population diversity of the songs. Brown et al. [23] performed a similar analysis on 220 songs from nine indigenous populations of Taiwan; in addition, they compared patterns of population-level diversity in songs, genes, and languages, and found that the pattern of musical diversity resembles more genetic than linguistic diversity.

Le Bomin et al. [25] argued that vertical transmission plays a major role in the formation of population-level musical diversity on the basis of their finding of a strong phylogenetic signal in a sample of 700 musical pieces collected in Gabon. In particular, they reported a delta score, a measure of deviation from treelike structure calculated from distance data [28, 29], of 0.29, which is comparable with those found in Indo-European languages (delta = 0.23, [9]) and Ainu languages (delta = 0.25, [30]), signaling a treelike structure inherent in the data. This result is in contrast to the aforementioned study on musical diversity in Austronesian-speaking populations, which suggested a greater departure from a treelike structure (delta = 0.46, [22]), indicative of horizontal transmission. Le Bomin et al. [25] further suggested that some character categories, such as rhythmic cells, metrics, and scales, exhibited a stronger phylogenetic signal than other categories.

Some songs are sung in specific contexts: work songs, hymns, and lullabies to mention a few. Some social contexts are common to various human societies, and acoustic features of songs predict their contexts even across different societies [18]. In ethnomusicology, songs are often categorized based on the social context. Considering the different functional roles that different songs may have played in modern and ancient human societies, it is plausible that cultural transmission of songs may depend on the context in which they are sung. To evaluate this, we conduct a largely exploratory study of traditional songs associated with different social contexts in the Ryukyu Archipelago of Japan.

The Ryukyu Archipelago is placed on the southwest part of Japan and composed of more than 150 islands, about 50 of which have residents. Since the islands have long been isolated by the sea, the Ryukyu Archipelago is known for its rich biodiversity and endemism [31–33]. The Ryukyuan population is genetically and morphologically differentiated from those in nearby regions, i.e., mainland Japan or Taiwan [34–42], and also genetically structured within the archipelago [43–45]. In terms of culture, Ryukyuan languages, which diverged from Japanese at least before the seventh century, can be divided into five subgroups [46]. They are spoken in distinct regions and differentiated from each other to the extent that they are often considered as independent languages rather than dialects [47].

The Ryukyu Archipelago is also known for its musical uniqueness. For example, a characteristic musical scale that is not seen in other part of Japan is widely shared within the archipelago [48, 49]. Common features of the music in the Ryukyu Archipelago include the presence of dance songs by members of community, the presence of songs by female religious leaders, and a high regard for the ability to sing and dance [49]. A stringed instrument called the sanshin is frequently used for accompaniment in every region of the archipelago, indicating the influence of the Ryukyu Kingdom, which was centered on Okinawa island from the 15th to 19th centuries. However, each region has unique cultural elements that are thought to have been originated before then especially in religious songs and songs for community events [50, 51]. In sum, these features seem to make the Ryukyu Archipelago an ideal field to study population-level musical diversity.

## Materials and methods

### Data

We used published musical scores of 1,342 traditional vocal songs in "A Survey of Japanese Folksongs–Okinawa-Amami Islands," compiled by Nippon Hoso Kyokai (NHK) [49]. The scores had been made from recordings collected during the period of 1962–1991 in the Ryukyu Archipelago of Japan with an intention to select songs reflecting traditional life and culture of each region and to include various types of songs. As Koizumi [48] pointed out, while folk songs change their forms as they are transmitted among people, art songs have normative forms and do not change during transmission. Popular songs, unlike folk songs, have no regional differences because they are transmitted rapidly and broadly. Therefore, for the purpose of this study, which is to analyze cultural transmission between populations, we focused only on traditional folk songs, excluding a small number of art songs and popular songs that are collected in the book for comparison with folk songs.

**Fig 1:**
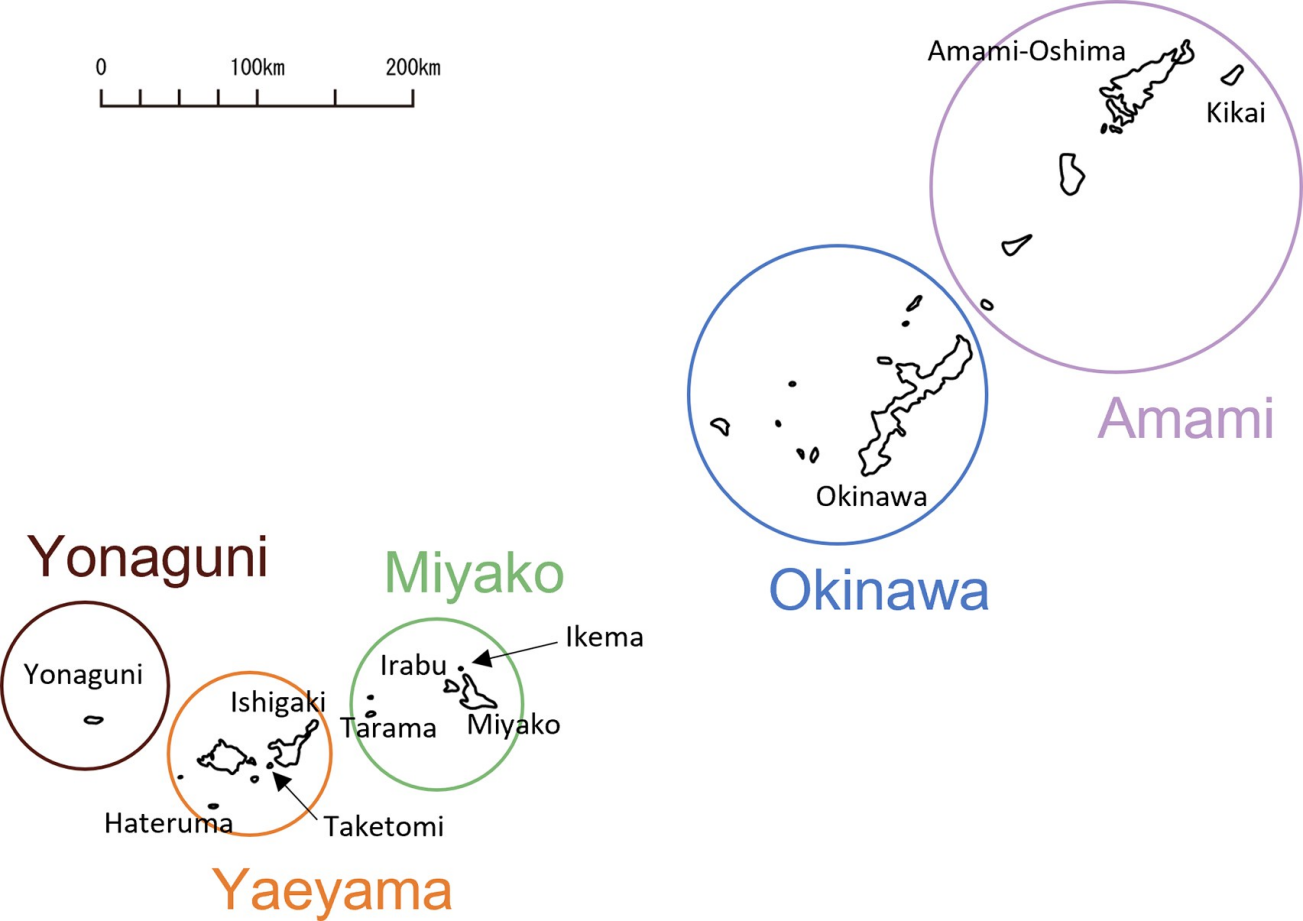
Map of the Ryukyu Archipelago. Five languages (dialects) are spoken in five regions (Amami, Okinawa, Miyako, Yaeyama and Yonaguni). The locations of the eleven islands for which either or both linguistic and genetic data were available are also indicated. Created based on a map from https://www.freemap.jp/ under a CC BY license, with permission from Keisuke Inoue, original copyright 2014.

Five geographical regions are recognized within the Ryukyu Archipelago, in each of which a distinct language, or dialect, is spoken, namely, the Amami, Okinawa, Miyako, Yaeyama, and Yonaguni regions (Fig 1) [46, 49]. Geographically, Yonaguni island is usually included in Yaeyama islands; however, in this study, we adopted the linguistic classification emphasizing the distinctiveness of the Yonaguni dialect [49]. Researchers had classified the songs into four groups on the basis of the social context in which they had been sung and referred to them as "child," "ritual," "work," and "amusement" songs (Table 1) [49]. The rationale for the categorization had been based on the situations of typical life in village communities of the Ryukyu Archipelago and in part derived from Yanagita [52].

**Table 1:** Numbers of songs used for the analysis in the five regions of the Ryukyu Archipelago and the eleven islands for which linguistic and/or genetic data were available.

Region	Island	Social context				Total
		Child	Ritual	Work	Amusement	
Amami		85	144	30	104	363
	Kikai	4	5	9	3	21
	Amami-Oshima	57	75	10	39	181
	Others	24	64	11	62	161
Okinawa		73	215	4	51	343
	Okinawa	57	160	3	10	230
	Others	16	55	1	41	113
Miyako		55	136	23	53	267
	Miyako	28	87	4	33	152
	Ikema	4	15	8	5	32
	Irabu	9	5	1	11	26
	Tarama	14	21	8	3	46
	Others	0	8	2	1	11
Yaeyama		35	158	65	64	322
	Ishigaki	9	36	38	7	90
	Taketomi	16	11	7	13	47
	Hateruma	2	13	1	3	19
	Others	8	98	19	41	166
Yonaguni	Yonaguni	6	13	4	24	47
Total		254	666	126	296	1342

Since neither linguistic nor genetic data existed in the islands lumped together in "others," songs from these islands were excluded from the analysis by island, although they were included in the analysis by region.

All songs were coded for the 26 musical characteristics (e.g., meter, tonality, melisma, and so on; S1 Appendix) of the CantoCore song classification scheme [26] by one of the authors (Y. Nishikawa). For example, CantoCore variable #9, "mode," is related to musical scales [26] (S1 Appendix). The Ryukyu scale, one of the frequent scales in the Ryukyu Archipelago [49], contains a pitch class at major third above the tonic and does not contain minor third notes, and songs with this scale are classified into "major iso-modal." On the other hand, the ritsu scale, another frequent scale in the archipelago, contains neither major nor minor third notes, and songs with this scale are classified into "a-modal." It should be noted that 15 performance-style variables from Cantometrics [20] were also coded in a previous study in Taiwan [23], but not adopted in this study because musical scores do not contain contextual information and the variables regarding performance-style cannot be coded from the scores.

### Musical distance

Using the algorithm described by Rzeszutek et al. [22], we obtained the distance between each pair of 1,342 songs based on the dissimilarity between songs in the codings for the 26 CantoCore structural variables. Since there is no a priori basis for deciding the relative contribution of each variable to the distance measure, all the variables were weighted equally, which facilitates comparison with previous studies. The distances among songs were visualized by multi-dimensional scaling (MDS) using R version 3.6.1.

We also quantified musical distances among the above-mentioned five regions and among selected islands within the Ryukyu Archipelago (see below) on the basis of the dissimilarity in songs between each pair of regions/islands. More specifically, pairwise *Φ*_ST_ values (described below) between regions/islands were used as musical distances, where all negative *Φ*_ST_ values, which occurs when the observed within-population variance exceeds between-population variance, were set to zero following Meirmans [53] and Rzeszutek et al. [22]. Note that here the unit of analysis is a population rather than a song, where we investigate on the patterns of musical diversity between and within populations. Based on the musical distances, which were normalized to an average distance of 1 as Gray et al. [9], Rzeszutek et al. [22], and Brown et al. [23], Neighbor-Net networks [54] were generated with SplitsTree4 [55] to illustrate the relationships among regions/islands. For each network, the delta score [28] was obtained to assess the treelikeness of the data.

### Local diversification

To investigate whether songs are differentiated among regions or islands, we ran analysis of molecular variance (AMOVA; [27]) on the distance matrix of the songs, where the extent of differentiation among regions/islands was measured by the *Φ*_ST_ statistics. Statistical significance of the *Φ*_ST_ value was evaluated under 1,000 times of random permutation of individual songs across populations regardless of their original population. We also obtained the pairwise *Φ*_ST_ value to measure the extent of diversification between a pair of islands. AMOVA was performed using pegas package of R version 3.6.1.

### Associations among songs, languages, and genes

Lexical data about the presence/absence of 675 cognate sets of ten islands (Amami-Oshima island in the Amami region, Okinawa island in the Okinawa region, Miyako, Ikema, Irabu, and Tarama islands in the Miyako region, Ishigaki, Taketomi, and Hateruma islands in the Yaeyama region, and Yonaguni island in the Yonaguni region) were obtained from Lee and Hasegawa [56], and the Jaccard distances, a measure of dissimilarity between two sets based on the Jaccard index [57], among the islands were calculated using vegan package of R version 3.6.1. Due to data availability, the linguistic distance was investigated only for the above ten islands.

337 bp of mtDNA HV-1 sequence data of five islands (Kikai (*n* = 24) and Amami-Oshima islands (*n* = 78) in the Amami region, Okinawa island (*n* = 95) in the Okinawa region, Miyako island (*n* = 66) in the Miyako region, and Ishigaki island (*n* = 63) in the Yaeyama region) were obtained from Matsukusa et al. [41] and Nishikawa and Ishida [45], and the pairwise *Φ*_ST_ values among the islands were calculated by AMOVA under the Tamura-Nei model [58] with gamma distribution using Arlequin ver 3.5.2.2 [59]. Due to data availability, the genetic distance was examined only for the above five islands. All negative *Φ*_ST_ values were set to zero.

We investigated how the musical diversity (i.e., the pairwise *Φ*_ST_ values) may be associated with the linguistic diversity (i.e., the Jaccard distances) and the genetic diversity (i.e., the pairwise *Φ*_ST_ values) among islands. Correlation between the distance matrices for songs and languages over the ten islands for which lexical data were available was examined by means of the Mantel test [60]. In the same way, correlation between the distance matrices for songs and genes over the five islands for which genetic data were available was examined. In addition, we ran partial Mantel tests [61] controlling for the geographic (great-circle) distances measured using Google Earth. The analysis was repeated for all songs pooled and separately for each of the four groups of songs with different social contexts (Table 1). Mantel and partial Mantel tests were performed using vegan package of R version 3.6.1.

## Results

### Musical distance

**Fig 2:**
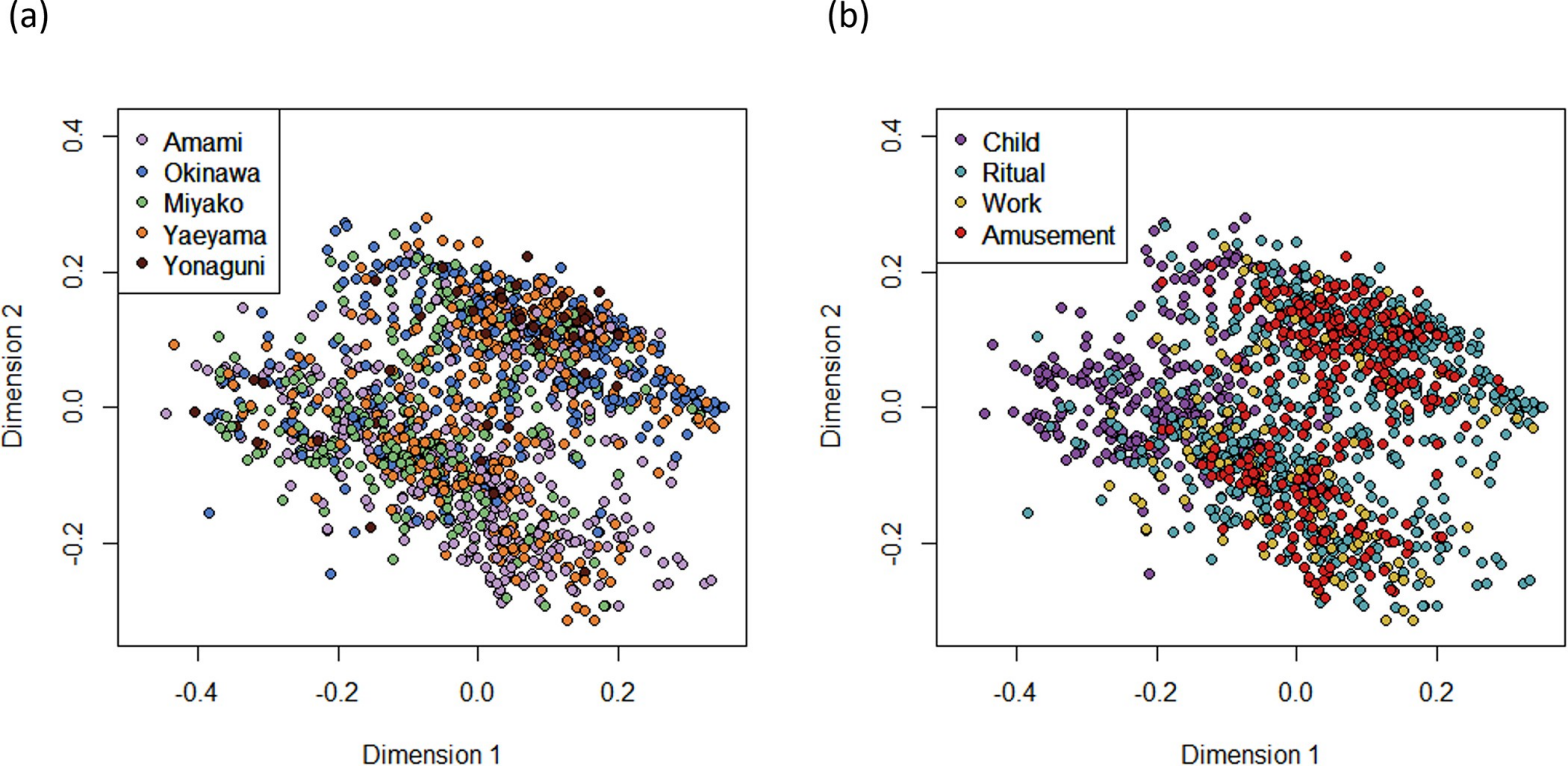
The multi-dimensional scaling (MDS) plots for 1,342 traditional vocal songs in the Ryukyu Archipelago. Colors indicate (a) the regions and (b) the social contexts.

The multi-dimensional scaling (MDS) plots for 1,342 traditional songs in the Ryukyu Archipelago are shown in Fig 2. Neither the songs from the same geographic regions (Fig 2A) nor those associated with the same social contexts (Fig 2B) formed clear clusters. While two clusters are discernible in Fig 2, these are unrelated to geography or social context, and roughly correspond to the difference between a-modal (bottom-left) and major iso-modal (top-right) songs (for details of CantoCore variables see Savage et al. [26]). Note that the former included the ritsu scale broadly used in East Asia including the Ryukyu Archipelago, and the latter included the Ryukyu scale used in the Ryukyu Archipelago and Indonesia [48].

**Fig 3:**
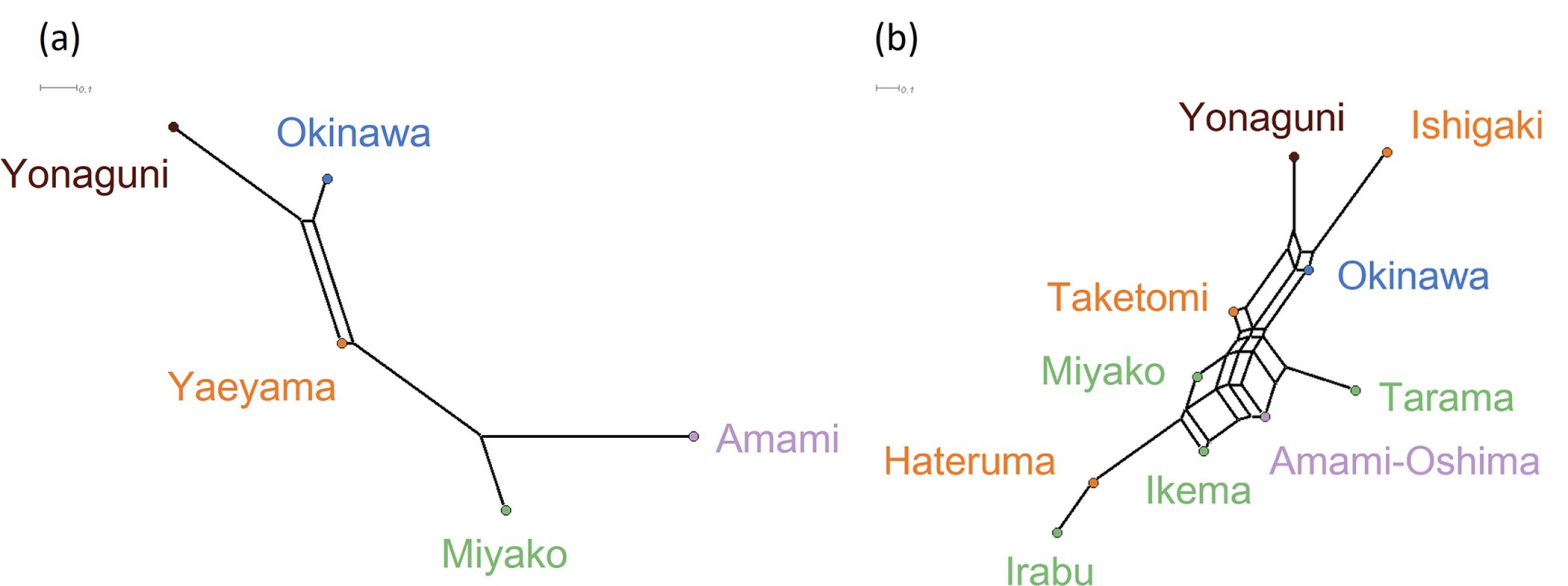
Neighbor-Net graphs. (a) Neighbor-Net graph based on the musical distances between 1,342 songs of the five regions in the Ryukyu Archipelago (*δ* = 0.346). (b) Neighbor-Net graph based on the musical distances between 870 songs of the ten islands for which linguistic data were available (*δ* = 0.456). Colors indicate the regions.

A Neighbor-Net graph based on the musical distances among the five regions in the Ryukyu Archipelago is shown in Fig 3A. Comparing Fig 3A with Fig 1, it was suggested that proximity in songs is not fully explicable by spatial proximity. Fig 3B shows a similar graph among the ten islands for which lexical data were available. The graph exhibited considerable reticulation, and islands in the same geographic regions did not form clusters. This is in contrast to a Neighbor-Net graph of the same ten islands generated from the linguistic distances (S1A Fig), which showed a treelike structure and clear clusters of spatially close islands, signaling the underlying phylogenetic relationships. A Neighbor-Net graph based on the genetic distances among the five islands (S1B Fig) showed that the topology of the graph was not consistent with the geography.

The delta scores for the musical networks of the five regions were 0.35 for all songs pooled and 0.49–0.68 when songs with different social contexts were considered separately (Table 2). The delta scores for the musical networks among the ten islands were 0.46 for all songs and 0.47–0.61 for each social context (Table 3). All the delta scores obtained for musical networks were larger than the delta score for the network of the ten islands based on the linguistic distance, 0.20. This indicated that musical networks are less treelike than linguistic networks, a possible interpretation of which is that horizontal transmission between islands has played a greater role in cultural evolution of music than that of language.

**Table 2:** *Φ*_ST_ and *δ* values among the five regions of the Ryukyu Archipelago (Amami, Okinawa, Miyako, Yaeyama, and Yonaguni regions) for songs associated with each social context.

Social context	*Φ*_ST_	*δ*
Child	0.006 (*p* < 0.001)	0.682
Ritual	0.006 (*p* < 0.001)	0.493
Work	0.033 (*p* < 0.001)	0.490
Amusement	0.014 (*p* < 0.001)	0.510
All social contexts	0.002 (*p* < 0.001)	0.347

*P*-values of *Φ*_ST_ are the probabilities of having a larger *Φ*_ST_ value than the observed value by random permutations.

**Table 3:** *Φ*_ST_ and *δ* values among ten islands in the Ryukyu Archipelago (Amami-Oshima, Okinawa, Miyako, Ikema, Irabu, Tarama, Ishigaki, Taketomi, Hateruma, and Yonaguni islands) for songs associated with each social context.

Social context	*Φ*_ST_	*δ*
Child	0.007 (*p* < 0.001)	0.606
Ritual	0.012 (*p* < 0.001)	0.509
Work	0.058 (*p* < 0.001)	-
Amusement	0.038 (*p* < 0.001)	0.469
All social contexts	0.003 (*p* < 0.001)	0.456

The delta score for "work" songs was not calculated because only one song of this category was available in each of Irabu and Hateruma islands and hence the pairwise *Φ*_ST_ could not be obtained between these islands. *P*-values of *Φ*_ST_ are the probabilities of having a larger *Φ*_ST_ value than the observed value by random permutations.

### Local diversification

The *Φ*_ST_ values for songs among the five regions and among the ten islands, calculated by AMOVA, are shown in Tables 2 and 3, respectively. The *Φ*_ST_ values for all songs pooled were 0.002 among the five regions and 0.003 among the ten islands, both of which were statistically significant (*p* < 0.001). These values were lower than the *Φ*_ST_ values for the songs among Austronesian-speaking populations (0.021, [22]) and among indigenous populations of Taiwan (0.047, [23]), suggesting that Ryukyuan songs as a whole is locally less diversified than songs in Austronesian-speaking populations and indigenous populations of Taiwan. However, it should be noted that 15 variables regarding performance-style from Cantometrics were added to the analyses in the previous study in Taiwan [23].

However, by analyzing the songs with different social contexts separately, it was indicated that the *Φ*_ST_ values vary depending on the social contexts (Tables 2 and 3). Especially, "work" songs had high *Φ*_ST_ values (0.033 among the five regions and 0.058 among the ten islands), indicating that songs sung in work-related contexts tend to be locally more diversified among populations than songs associated with other social contexts. A bootstrap analysis, where *Φ*_ST_ values calculated for 1,000 bootstrap samples were compared between different social contexts, suggested that the *Φ*_ST_ value for "work" songs was significantly larger than those for songs associated with other social contexts (S2 Fig).

### Associations among songs, languages, and genes

**Fig 4:**
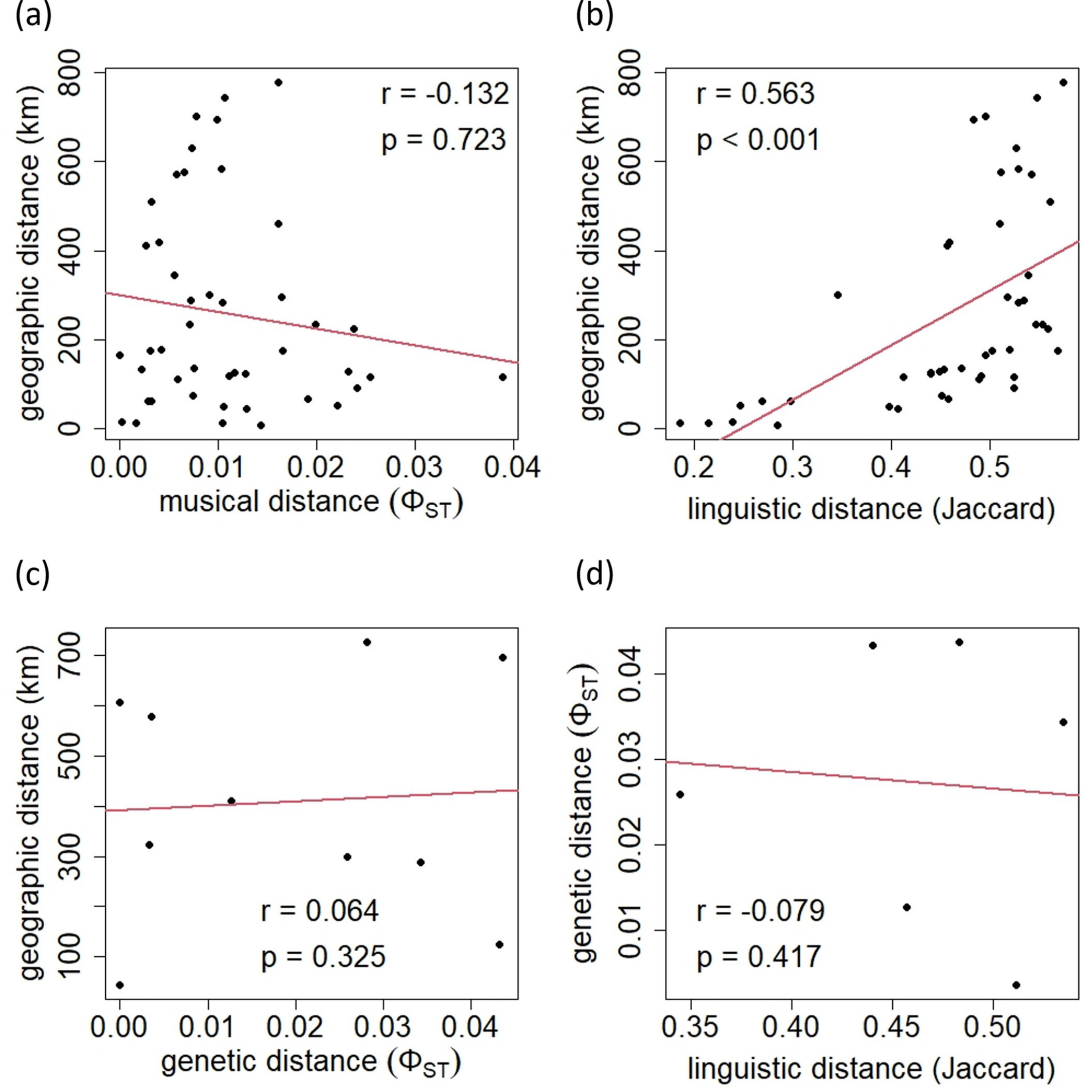
Regression plots. Regression plots for (a) music versus geography in the ten islands for which linguistic data were available, (b) languages versus geography in the ten islands, (c) genes versus geography in the five islands for which genetic data were available, and (d) languages versus genes in the four islands for which both linguistic and genetic data were available. There was significant correlation between languages and geography.

Fig 4A plots the geographic distances among all pairs from the ten islands for which lexical data were available against the pairwise *Φ*_ST_ values for all songs. A Mantel test did not detect a statistically significant correlation between music and geography (*r* = −0.132, *p* = 0.723). In Fig 4B, the same geographic distances are plotted against the Jaccard distances for languages, showing a significant positive correlation between language and geography (*r* = 0.563, *p* < 0.001). Fig 4C shows a similar relationship between geographic distances and the pairwise *Φ*_ST_ values for the mtDNA sequence among the five islands for which genetic data were available, suggesting no correlation (*r* = 0.064, *p* = 0.325). Significant correlation was not observed either between languages and genes among four islands for which both linguistic and genetic data were available (Fig 4D, *r* = −0.079, *p* = 0.417).

**Fig 5:**
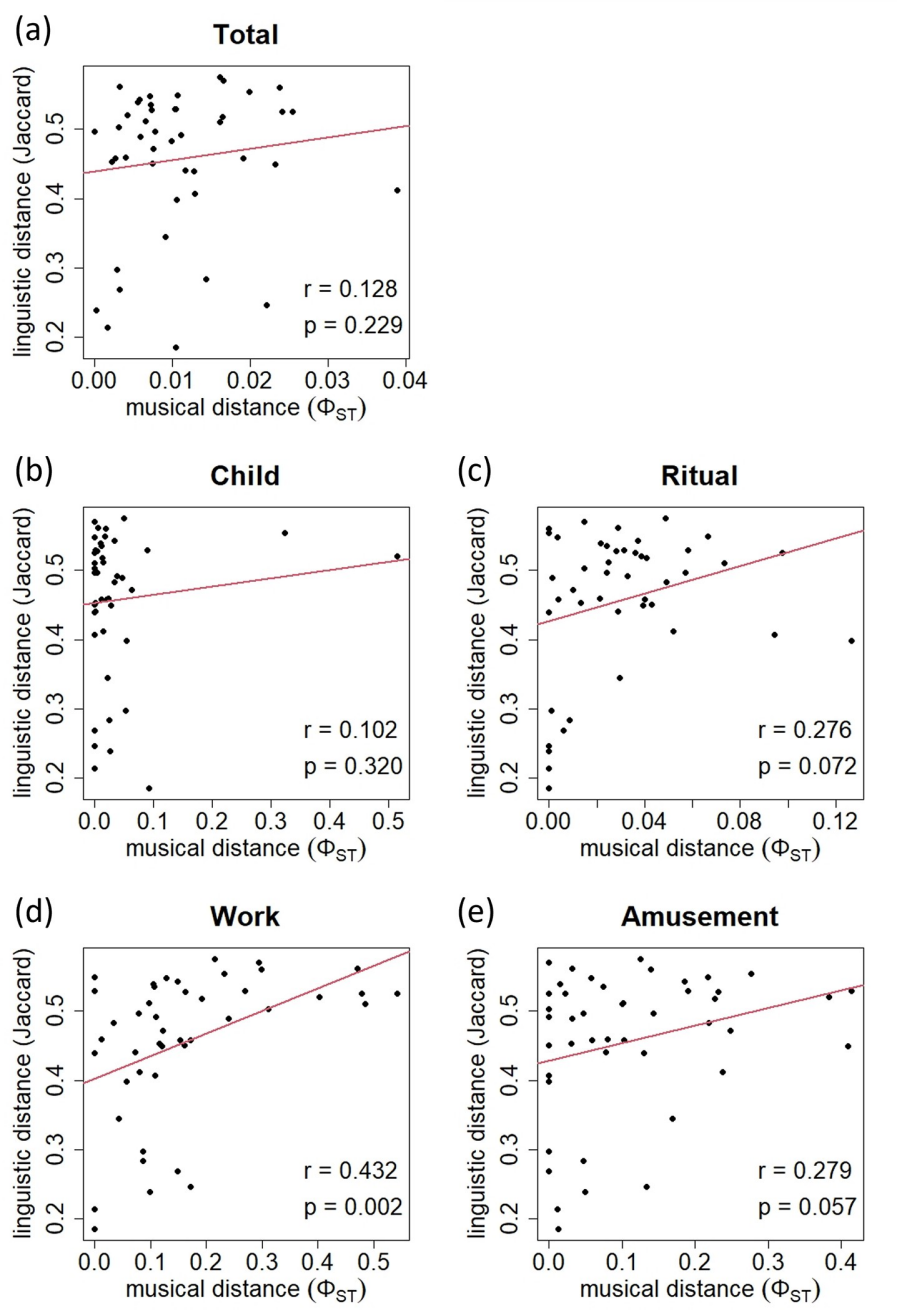
Regression plots. Regression plots for songs of (a) all social contexts pooled, (b) "child," (c) "ritual," (d) "work," and (e) "amusement" versus languages in the ten islands for which linguistic data were available. There was significant correlation between songs of "work" and languages.

Fig 5 shows the relationship between the musical and linguistic distances among the ten islands for which lexical data were available for all songs pooled (Fig 5A) and for each group of songs sharing the same social context (Fig 5B–5E). According to Mantel tests, statistically significant correlation was found only for "work" songs (Fig 5D, *r* = 0.432, *p* = 0.002). Since the linguistic distance was shown to be correlated with the geographic distance (see Fig 4B), we also examined correlation between songs and languages after controlling for geography. The results of partial Mantel tests indicated that the musical distances for "work" songs were positively correlated with the linguistic distance among the ten islands when the geographic distance was statistically controlled for (Table 4).

**Table 4:** Full and partial correlation coefficients between musical and linguistic distances among ten islands (Amami-Oshima, Okinawa, Miyako, Ikema, Irabu, Tarama, Ishigaki, Taketomi, Hateruma, and Yonaguni islands) and between musical and genetic distances among five islands (Kikai, Amami-Oshima, Okinawa, Miyako, and Ishigaki islands) in the Ryukyu Archipelago.

Social context	Correlation between musical and linguistic distances		Correlation between musical and genetic distances	
	*r* (full)	*r* (partial)	*r* (full)	*r* (partial)
Child	0.102 (*p* = 0.320)	0.167 (*p* = 0.165)	0.003 (*p* = 0.500)	−0.052 (*p* = 0.525)
Ritual	0.276 (*p* = 0.072)	0.178 (*p* = 0.182)	0.283 (*p* = 0.242)	0.309 (*p* = 0.233)
Work	0.432 (*p* = 0.002)	0.548 (*p* < 0.001)	−0.550 (*p* = 0.933)	−0.585 (*p* = 0.933)
Amusement	0.279 (*p* = 0.057)	0.127 (*p* = 0.255)	−0.012 (*p* = 0.517)	−0.022 (*p* = 0.533)
All social contexts	0.128 (*p* = 0.229)	0.247 (*p* = 0.066)	0.181 (*p* = 0.342)	0.174 (*p* = 0.350)

Partial correlation coefficients were obtained after controlling for the geographic distance.

Similarly, we investigated the association between the musical and genetic distances among the five islands for which genetic data were available for all songs pooled and for each group of songs having the same social context. No significant correlation was found by Mantel tests. Neither did partial Mantel tests detect any significant correlation between songs and genes after controlling for geography (Table 4).

Finally, we made an additional investigation on "child" songs, which were further subdivided into "children’s songs" (*n* = 175) and "lullabies" (*n* = 27). Children’s songs were songs sung by children, while lullabies were songs sung toward children. Hence, it was possible that these two groups of songs may be transmitted in different ways. More specifically, we predicted that lullabies, but not necessarily children’s songs, tend to be transmitted from mothers to children in the same manner as mtDNA, and thus there is an association between them. This prediction was, however, not supported by Mantel or partial Mantel tests for correlation between lullabies and mtDNA sequences (Table 5).

**Table 5:** Full and partial correlation coefficients between musical and linguistic distances and between musical and genetic distances among ten and five islands, respectively (see Table 4) for children’s songs and lullabies, which are subsets of the "child" songs.

	Correlation between musical and linguistic distances		Correlation between musical and genetic distances	
	*r* (full)	*r* (partial)	*r* (full)	*r* (partial)
Children’s songs	0.136 (*p* = 0.242)	0.253 (*p* = 0.096)	0.128 (*p* = 0.383)	0.114 (*p* = 0.392)
Lullabies	0.141 (*p* = 0.268)	0.144 (*p* = 0.263)	−0.306 (*p* = 0.833)	−0.340 (*p* = 0.875)

Partial correlation coefficients were obtained after controlling for the geographic distance.

## Discussion

Both Neighbor-Net graphs and the delta scores suggested that musical networks of traditional songs in the Ryukyu Archipelago are less treelike than the linguistic network. A possible interpretation is that horizontal transmission between islands may have played a greater role in the formation of musical diversity than that of linguistic diversity. Deviation from treelike structure could also occur as a result of convergent evolution; however, evidence for association between song similarity and environmental or lifestyle similarity across islands, which would support this possibility, is thus far lacking.

The network based on linguistic distances showed clear clusters of spatially close islands (S1B Fig), which may reflect vertical transmission of languages associated with the history of population divergence. Linguistic distance was also positively correlated with geographic distance between islands (Fig 4B). On the other hand, such correspondence with geography was not observed in the musical or genetic networks (S1A, S1C Fig, Fig 4A and 4C). This may be due to horizontal transmission between relatively distant islands. As a recent genomic analysis has suggested [44], there may have been undocumented small-scale migration events between islands within the Ryukyu Archipelago. Since the difficulty of an inter-island voyage depends not only on the geographic distance, but also on other factors such as tidal currents, these migration events may have attenuated the correlation between genetic and geographic distances. It is also plausible that songs culturally diffused through such migration events as well as during social interactions between the residents of different islands, the latter of which were likely contingent on economic and political factors. For example, the influence of Okinawa island, the largest island in the archipelago, may be the reason of the relatively small pairwise *Φ*_ST_ values for music between Okinawa island and the other islands (Fig 3B, S2 Data). Conversely, those migrations and social interactions do not seem to have massively altered the basic vocabulary of each language in the Ryukyu Archipelago, perhaps because the conformity effect, by which children tend to acquire cultural variants used by the majority of people, played a greater role in the learning of basic vocabulary than that of songs.

It is thus far difficult to connect the topologies of musical or genetic networks with known history of the area. For example, Amami-Oshima and Miyako islands, which are located close to each other in the genetic network (S1B Fig), are geographically separated and no historical records linking them have been recognized. For more detailed discussion, studies using whole genome in a large number of islands are awaited. While we were able to analyze large amount of musical data covering many islands, linguistic data were available only for a part of those islands, and scarcity is even more acute for genetic data. Furthermore, considering lexicon and phonology show quite different networks in Northeast Asia [62], the use of phonological data in addition to the lexical data may affect our results. Future research should take these issues into consideration.

The extent of song diversification among populations in the Ryukyu Archipelago is smaller than that of Taiwan indigenous populations. However, the observed *Φ*_ST_ values indicated that the extent of song diversification among regions/islands may vary depending on the social contexts associated with the songs; that is, the social context may have an effect on how songs are transmitted between and within populations. This is consistent with the previous argument that patterns of transmission vary across cultural domains [7, 8, 10, 63–66]. In particular, the "work" songs exhibited high degree of between-region diversity compared to songs with other social contexts.

Association between the songs and mtDNA was not observed in the Ryukyu Archipelago, which is in contrast to the observed association between songs and mtDNA in Taiwan [23]. A possible reason for the discrepancy is that the association is weaker in the Ryukyuan people because they are genetically and culturally less diversified than Taiwan indigenous populations. While AMOVA for the five Ryukyuan islands obtained a statistically significant *Φ*_ST_ value (*Φ*_ST_ = 0.022, *p* < 0.001), the extent of between-population genetic differentiation is much lower than for the nine Taiwanese populations (*Φ*_ST_ = 0.127, [23]). In fact, the Taiwanese indigenous populations are known to have striking genetic diversity between each other, presumably because of long-term social isolation and endogamy [67–69]. As for the Ryukyu Archipelago, on the other hand, where the present-day inhabitants are originated from migrations in the Holocene with little or no genetic contribution by the Pleistocene inhabitants [43, 70], the whole archipelago became a single cultural zone with exchanges and migrations between islands since the eleventh or twelfth century [44, 71].

In addition, the hypothesis that lullabies tend to be maternally transmitted was not supported by our mtDNA data. It is possible that the assumption that lullabies are vertically transmitted from mothers to children is false. In other words, lullabies are sometimes sung by baby-sitters other than mothers [49], and children may not necessarily learn the lullabies they are exposed to by their mothers. It should be noted, however, that we used mtDNA data from only five islands, and thus further analysis with extended data might obtain different results.

The social bonding hypothesis for the evolution of musicality states that music has been adaptive because it enhances social bonding and cooperative behavior within group [72–77] as has been supported by several empirical studies [78–82]. There exists evidence suggesting that singing induces the elevation of oxytocin concentration [83, 84] and the release of endorphins [81, 82, 85]. Oxytocin and endorphins (or more generally, the endogenous opioid system, EOS) may be part of physiological mechanisms that enhance social bonding in humans (reviewed in Savage et al. [77]). It has also been suggested that EOS is associated with social bonding in non-human primates [86–90] and other animals [91–93]. Therefore, singing together in a group is thought to strengthen social bonding. It might be argued that our observation of large *Φ*_ST_ value for "work" songs and statistically significant association between "work" songs and language are in line with the social bonding hypothesis, because singing during working may enhance social bonding of the collaborative team and increase work efficiency. This may be of particular adaptive value in food production like farming or fishing. To sing together, members of a team have to know the same song repertoires, and this may limit changes in songs either by innovation or horizontal transmission, leading to small within-group and large between-group divergence. While singing together may also promote social bonding in other contexts, the effect may be particularly important in joint works requiring precise coordination, and as a consequence, "work" songs may be functionally more restricted than other songs.

In conclusion, horizontal transmission may have played a large role in song evolution in the Ryukyu Archipelago. The extent of song diversification varies depending on the social contexts in which songs are sung, and this indicates the importance of considering social context in studying cultural evolution of music. "Work" songs exhibited high degree of between-region diversity and association with languages, partially congruent with the social bonding hypothesis for the evolution of musicality.

## Supporting information

S1 Data Cantocore codings of 1,342 songs. Each row represents a song and the first column corresponds to the song ID in "A Survey of Japanese Folksongs–Okinawa-Amami Islands." The second, third, and fourth columns represent the region, island, and social context of each song. The fifth and later columns represent the coding for each structural variable of the CantoCore song classification scheme.
(CSV)

S2 Data CSV files of the musical, linguistic, genetic, and geographic distances between the islands and NEXUS files for the Neighbor-Net analysis with SplitsTree4. (ZIP)

S1 Appendix Description of 26 structural variables of the CantoCore song classification scheme by Savage et al. (2012). For more information, see Savage et al. (2012).
(PDF)

S2 Appendix R source code for calculating the distances between songs, performing MDS, performing AMOVA, and performing Mantel and partial Mantel tests. (PDF)

S1 Fig Neighbor-Net graphs. (a) Neighbor-Net graph based on the linguistic distances among the ten islands (*δ* = 0.202). (b) Neighbor-Net graph based on the genetic distances among the five islands (*δ* = 0.193). Colors indicate the regions.
(PDF)

S2 Fig Comparison of *Φ*_ST_ values between social contexts. Each panel shows the frequency distribution of the difference in the *Φ*_ST_ values for the five regions (Fig 1) between different social contexts calculated from 1,000 bootstrap samples. The observed differences in *Φ*_ST_ are indicated by the vertical dashed lines. *Φ*_ST_ of "work" songs was significantly larger than that of (a) "child" songs (*p* = 0.023), (b) "ritual" songs (*p* = 0.023), and (c) "amusement" songs (*p* = 0.012).
(PDF)
