# Supplementary material for: Cultural transmission of traditional songs in the Ryukyu Archipelago
Source: PLoS One. 2022 Jun 24;17(6):e0270354. doi: 10.1371/journal.pone.0270354 (PMC9231793; doi:10.1371/journal.pone.0270354)
Supplement: S1 Appendix — For more information, see Savage et al. (2012). (PDF) [file pone.0270354.s003.pdf]

S1 Appendix. Description of 26 structural variables of the CantoCore song classification scheme by Savage et al. (2012). For more information, see Savage et al. (2012).

1) Meter
   (a) A-metric
   (b) Hetero-metric
   (c) Poly-metric
   (d) Iso-metric

2) No. of beats
   (a) Duple
   (b) Triple
   (c) Complex
   (n/a) A-/hetero-/poly-metric

3) Beat sub-division
   (a) A-divisive
   (b) Hetero-divisive
   (c) Iso-divisive
   (n/a) A-/hetero-/poly-metric

4) No. of sub-beats
   (a) Simple
   (b) Compound
   (c) Complex
   (n/a) A-/hetero-/poly-metric or a-/hetero-/poly-divisive

5) Syncopation
   (0) Little or no syncopation
   (0.5) Moderately syncopated
   (1) Highly syncopated
   (n/a) A-/hetero-/poly-metric

6) Motivic redundancy
   (0) Non-motivic
   (0.5) Moderately motivic
   (1) Highly motivic

7) Durational variability
   (0) Low durational variability
   (0.5) Moderate durational variability
   (1) High durational variability

8) Tonality
   (a) Indeterminate a-tonal
   (b) Discrete a-tonal
   (c) Hetero-tonal
   (d) Poly-tonal
   (e) Iso-tonal

9) Mode

(a) A-modal

(b) Hetero-modal

(c) Poly-modal

(d) Minor iso-modal

(e) Major iso-modal

(n/a) A-/hetero-/poly-tonal

10) No. of scale degrees

(0) Sparse scale

(0.5) Moderately dense scale

(1) Dense scale

(n/a) A-/hetero-/poly-tonal

11) Hemitonicity

(0) Anhemitonic

(0.5) Moderately hemitonic

(1) Highly hemitonic

12) Melodic interval size

(0) Small intervals

(0.5) Medium intervals

(1) Large intervals

13) Melodic range

(0) Small range

(0.5) Medium range

(1) Large range

14) Melodic contour

(a) Horizontal

(b) Ascending

(c) Descending

(d) U-shaped

(e) Arched

(f) Undulating

15) Melisma

(0) Syllabic: 1–2 notes

(0.5) Mildly melismatic

(1) Strongly melismatic

16) Vocables

(0) Few vocables

(0.5) Some vocables

(1) Many vocables

17) No. of vocal parts

(0) One-part

(0.5) Two-part

(1) Many-part

18) Rhythmic texture

(a) Hetero-rhythmic (heterophonic)

(b) Poly-rhythmic (polyphonic)

(c) Iso-rhythmic (homophonic)

(n/a) One-part (monophonic)

19) Harmonic texture

(0) Rough (“dissonant”)

(0.5) Smooth (“consonant”)

(n/a) One-part (includes 0–49 and 1150–1200 cents), or poly-/hetero-rhythmic

20) Relative motion

(a) Hetero-contour (drone)

(b) Poly-contour (independent motion)

(c) Iso-contour (parallel motion)

(n/a) One-part

21) Phrase repetition

(0) Non-repetitive

(0.5) Moderately repetitive

(1) Repetitive

22) Phrase length

(0) Short phrases

(0.5) Medium-length phrases

(1) Long phrases

23) Phrase symmetry

(0) Symmetric

(0.5) Mildly asymmetric

(1) Very asymmetric

24) Solo/group arrangement

(a) Solo

(b) Mixed

(c) Alternating

(d) Group

25) Responsorial arrangement

(a) A-responsorial

(b) Hetero-responsorial

(c) Iso-responsorial

26) Phrase overlap

(0) Non-overlapping

(0.5) Mildly overlapping

(1) Highly overlapping

(n/a) A-responsorial
