# Supplementary material for: Cultural transmission of traditional songs in the Ryukyu Archipelago
Source: PLoS One. 2022 Jun 24;17(6):e0270354. doi: 10.1371/journal.pone.0270354 (PMC9231793; doi:10.1371/journal.pone.0270354)
Supplement: S2 Appendix — (PDF) [file pone.0270354.s004.pdf]

S2 Appendix. R source code for calculating the distances between songs, performing MDS, performing AMOVA, and performing Mantel and partial Mantel tests.

1. R source code for calculating the distances between songs. Based on the code used in Rzeszutek et al. (2012) and Brown et al. (2014), available at https://github.com/pesavage/circumpolar-music/blob/master/AinuMusic.R.

```
ordinal.fn<-function (x,y) {
if(is.na(x)|is.na(y))"NA" else
abs(x-y)}
weightedv6.dist<-function(d,ord,nom) {
d[d==""]<-NA
nominal<-cbind(d[,nom])
nominal.fields<-vector("list",length=length(nominal[1,]))
for (i in 1:length(nominal[1,])) {nominal.fields[[i]]<-
matrix(nrow=length(nominal[,1]),ncol=13)}
for (i in 1:length(nominal[1,])) {rownames(nominal.fields[[i]])<-rownames(nominal)}
for (i in 1:length(nominal[1,])) {colnames(nominal.fields[[i]])<-
c("a","b","c","d","e","f","g","h","i","j","k","l","m")}
for (j in 1:13){
for (k in 1:length(nominal[,1])){
for (i in 1:length(nominal[1,])){nominal.fields[[i]][k,j]<-(if(is.na(nominal[k,i]))"NA"
else if(substr(nominal[k,i],1,1)==colnames(nominal.fields[[i]])[j] |
substr(nominal[k,i],2,2)==colnames(nominal.fields[[i]])[j]
|substr(nominal[k,i],3,3)==colnames(nominal.fields[[i]])[j]
|substr(nominal[k,i],4,4)==colnames(nominal.fields[[i]])[j]
|substr(nominal[k,i],5,5)==colnames(nominal.fields[[i]])[j]
|substr(nominal[k,i],6,6)==colnames(nominal.fields[[i]])[j]
|substr(nominal[k,i],7,7)==colnames(nominal.fields[[i]])[j]
|substr(nominal[k,i],8,8)==colnames(nominal.fields[[i]])[j]
|substr(nominal[k,i],9,9)==colnames(nominal.fields[[i]])[j]
|substr(nominal[k,i],10,10)==colnames(nominal.fields[[i]])[j]
|substr(nominal[k,i],11,11)==colnames(nominal.fields[[i]])[j]
|substr(nominal[k,i],12,12)==colnames(nominal.fields[[i]])[j]
|substr(nominal[k,i],13,13)==colnames(nominal.fields[[i]])[j])1 else 0) }}}
```

```
suppressWarnings(for (i in 1:length(nominal[1,])){storage.mode(nominal.fields[[i]])<-
"numeric"})
nominal.dist<-vector("list",length=length(nominal[1,]))
for (i in 1:length(nominal[1,])) {nominal.dist[[i]]<-
matrix(nrow=length(nominal[,1]),ncol=length(nominal[,1]))}
nominal.result<-
matrix(nrow=length(d[,1]),ncol=length(d[,1]),c(rep(0,(length(d[,1])*length(d[,1])))))
ordinal<-cbind(d[,ord])
suppressWarnings(storage.mode(ordinal)<-"numeric")
ordinal.dist<-vector("list",length=length(ordinal[1,]))
for (i in 1:length(ordinal[1,])) {ordinal.dist[[i]]<-
matrix(nrow=length(ordinal[,1]),ncol=length(ordinal[,1]))}
ordinal.result<-
matrix(nrow=length(d[,1]),ncol=length(d[,1]),c(rep(0,(length(d[,1])*length(d[,1])))))
result<-matrix(nrow=length(d[,1]),ncol=length(d[,1]))
for (k in 1:length(nominal[,1])){
for (j in 1:length(nominal[,1])){
for (i in 1:length(nominal[1,])){nominal.dist[[i]][k,j]<-
if(is.na(nominal.fields[[i]][k,1])|is.na(nominal.fields[[i]][j,1]))"NA" else
((if(nominal.fields[[i]][k,1]==nominal.fields[[i]][j,1])0 else
1)+(if(nominal.fields[[i]][k,2]==nominal.fields[[i]][j,2])0 else
1)+(if(nominal.fields[[i]][k,3]==nominal.fields[[i]][j,3])0 else
1)+(if(nominal.fields[[i]][k,4]==nominal.fields[[i]][j,4])0 else
1)+(if(nominal.fields[[i]][k,5]==nominal.fields[[i]][j,5])0 else
1)+(if(nominal.fields[[i]][k,6]==nominal.fields[[i]][j,6])0 else
1)+(if(nominal.fields[[i]][k,7]==nominal.fields[[i]][j,7])0 else
1)+(if(nominal.fields[[i]][k,8]==nominal.fields[[i]][j,8])0 else
1)+(if(nominal.fields[[i]][k,9]==nominal.fields[[i]][j,9])0 else
1)+(if(nominal.fields[[i]][k,10]==nominal.fields[[i]][j,10])0 else
1)+(if(nominal.fields[[i]][k,11]==nominal.fields[[i]][j,11])0 else
1)+(if(nominal.fields[[i]][k,12]==nominal.fields[[i]][j,12])0 else
1))/((if(nominal.fields[[i]][k,1]==1 | nominal.fields[[i]][[j,1]]==1)1 else
0)+(if(nominal.fields[[i]][k,2]==1 | nominal.fields[[i]][[j,2]]==1)1 else
0)+(if(nominal.fields[[i]][k,3]==1 | nominal.fields[[i]][[j,3]]==1)1 else
0)+(if(nominal.fields[[i]][k,4]==1 | nominal.fields[[i]][[j,4]]==1)1 else
0)+(if(nominal.fields[[i]][k,5]==1 | nominal.fields[[i]][[j,5]]==1)1 else
```

```
0)+(if(nominal.fields[[i]][k,6]==1 | nominal.fields[[i]][[j,6]]==1)1 else
0)+(if(nominal.fields[[i]][k,7]==1 | nominal.fields[[i]][[j,7]]==1)1 else
0)+(if(nominal.fields[[i]][k,8]==1 | nominal.fields[[i]][[j,8]]==1)1 else
0)+(if(nominal.fields[[i]][k,9]==1 | nominal.fields[[i]][[j,9]]==1)1 else
0)+(if(nominal.fields[[i]][k,10]==1 | nominal.fields[[i]][[j,10]]==1)1 else
0)+(if(nominal.fields[[i]][k,11]==1 | nominal.fields[[i]][[j,11]]==1)1 else
0)+(if(nominal.fields[[i]][k,12]==1 | nominal.fields[[i]][[j,12]]==1)1 else 0))}}}
suppressWarnings(for (i in 1:length(nominal[1,])){storage.mode(nominal.dist[[i]])<-
"numeric"})
vnom<-vector(mode="numeric",length=length(nominal[1,]))
for (k in 1:length(nominal[,1])){
for (j in 1:length(nominal[,1])){
for (i in 1:length(nominal[1,])){
   vnom[i]<-nominal.dist[[i]][k,j]
   }
   nominal.result[k,j]<-mean(suppressWarnings(as.numeric(vnom)),na.rm=TRUE)
   }}
for (k in 1:length(ordinal[,1])){
for (j in 1:length(ordinal[,1])){
for (i in 1:length(ordinal[1,])){ordinal.dist[[i]][k,j]<-
ordinal.fn(x=ordinal[k,i],y=ordinal[j,i]) }}}
suppressWarnings(for (i in 1:length(ordinal[1,])){storage.mode(ordinal.dist[[i]])<-
"numeric"})
vord<-vector(mode="numeric",length=length(ordinal[1,]))
for (k in 1:length(ordinal[,1])){
for (j in 1:length(ordinal[,1])){
for (i in 1:length(ordinal[1,])){
   vord[i]<-ordinal.dist[[i]][k,j]
   }
   ordinal.result[k,j]<-mean(suppressWarnings(as.numeric(vord)),na.rm=TRUE)
   }}
for (k in 1:length(nominal[,1])){
for (j in 1:length(nominal[,1])){
result[k,j]<-if(is.na(nominal.result[k,j]))ordinal.result[k,j] else
if(is.na(ordinal.result[k,j])) nominal.result[k,j] else (ordinal.result[k,j]*length(ord) +
nominal.result[k,j]*length(nom))/(length(ord)+length(nom))
```

```
}}
row.names(result)<-row.names(d)
colnames(result)<-row.names(d)
as.dist(result)
}
data<-as.matrix(read.csv("S1_Data.csv",header=TRUE,row.names=1))
songs<-data[,4:29]
labels<-data[,c(1,3)]
songs.dist<-
weightedv6.dist(songs,c(5:7,10:13,15:17,19,21:23,26),c(1:4,8:9,14,18,20,24:25))
```

2. R source code for performing MDS.

```
resultmds<-cmdscale(songs.dist,k=10,eig=T)
library(khroma)
col.pal<-colour("smooth rainbow")
cols<-col.pal(10)
labels<-gsub("Amami",2,labels)
labels<-gsub("Okinawa",4,labels)
labels<-gsub("Miyako",6,labels)
labels<-gsub("Yaeyama",8,labels)
labels<-gsub("Yonaguni",10,labels)
labels<-gsub("Child",3,labels)
labels<-gsub("Ritual",5,labels)
labels<-gsub("Work",7,labels)
labels<-gsub("Amusement",9,labels)
plot(resultmds$points[,1],resultmds$points[,2],xlab="Dimension 1",ylab="Dimension
2",main="",bg=as.vector(cols[as.numeric(labels[,1])]),pch=21,xlim=c(-
0.48,0.35),ylim=c(-0.32,0.41))
names<-c("Amami","Okinawa","Miyako","Yaeyama","Yonaguni")
cols2<-c(cols[2],cols[4],cols[6],cols[8],cols[10])
legend("topleft",legend=names,col=cols2,pch=16,bg="transparent")
legend("topleft",legend=names,pch=21,bg="transparent")
```

3. R source code for performing AMOVA. Example for evaluating the extent of diversification among the five regions for all songs.

```
library(pegas)
library(ade4)
d<-lingoes(songs.dist)
p<-factor(c(rep(1,363),rep(2,343),rep(3,267),rep(4,322),rep(5,47)))
result<-pegas::amova(d~p,nperm=1000,is.squared=FALSE)
sig2<-setNames(result$varcomp$sigma2,rownames(result$varcomp))
phi<-getPhi(sig2)
phi<-phi[1,1]
```

4. R source code for performing Mantel and partial Mantel tests. Example for examining correlation between music and languages.

```
x<-as.dist(read.csv("total10.csv",header=TRUE,row.names=1))
y<-as.dist(read.csv("language.csv",header=TRUE,row.names=1))
z<-as.dist(read.csv("geography10.csv",header=TRUE,row.names=1))
par(ps=20)
par(mar=c(5,5,4,2))
plot(x,y,xlab=expression(paste("musical distance ",{(Φ[ST])})),ylab="linguistic
distance (Jaccard)",pch=16,col=1,main="Total")
lm<-lm(y~x)
abline(lm,lwd=2,col=2)
library(vegan)
mantel(x,y,permutations=10000,na.rm=TRUE)
mantel.partial(x,y,z,permutations=10000,na.rm=TRUE)
legend("bottomright",legend=c("r = 0.158","","p = 0.179",""),box.lty=0)
```
