# Supplementary material for: Cultural transmission of traditional songs in the Ryukyu Archipelago
Source: PLoS One. 2022 Jun 24;17(6):e0270354. doi: 10.1371/journal.pone.0270354 (PMC9231793; doi:10.1371/journal.pone.0270354)
Supplement: S1 Fig — (a) Neighbor-Net graph based on the linguistic distances among the ten islands (δ = 0.202). (b) Neighbor-Net graph based on the genetic distances among the five islands (δ = 0.193). Colors indicate the regions. (PDF) [file pone.0270354.s005.pdf]

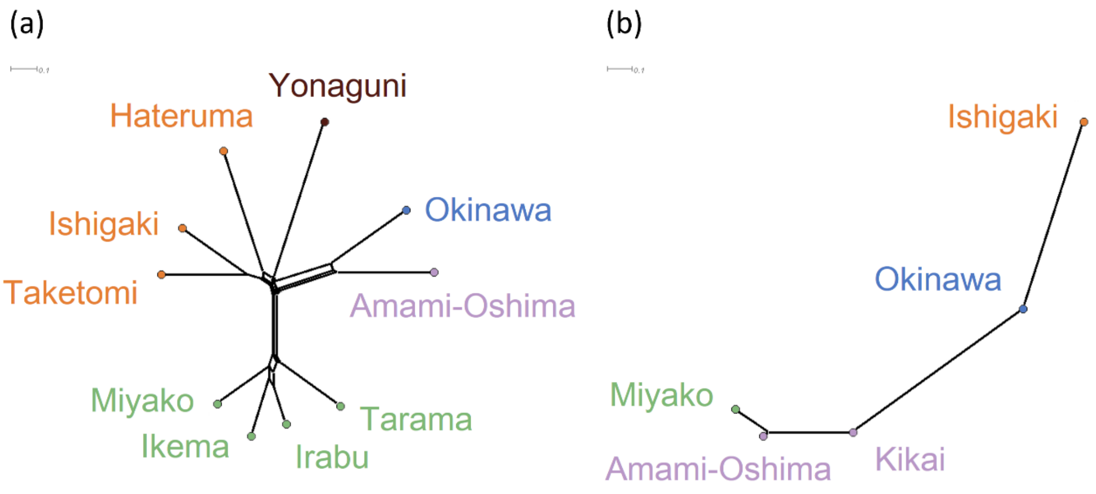

S1 Fig. Neighbor-Net graphs. (a) Neighbor-Net graph based on the linguistic distances among the ten islands ($\delta = 0.202$). (b) Neighbor-Net graph based on the genetic distances among the five islands ($\delta = 0.193$). Colors indicate the regions.
