# Supplementary material for: Cultural transmission of traditional songs in the Ryukyu Archipelago
Source: PLoS One. 2022 Jun 24;17(6):e0270354. doi: 10.1371/journal.pone.0270354 (PMC9231793; doi:10.1371/journal.pone.0270354)
Supplement: S2 Fig — Each panel shows the frequency distribution of the difference in the ΦST values for the five regions (Fig 1) between different social contexts calculated from 1,000 bootstrap samples. The observed differences in ΦST are indicated by the vertical dashed lines. ΦST of "work" songs was significantly larger than that of (a) "child" songs (p = 0.023), (b) "ritual" songs (p = 0.023), and (c) "amusement" songs (p = 0.012). (PDF) [file pone.0270354.s006.pdf]

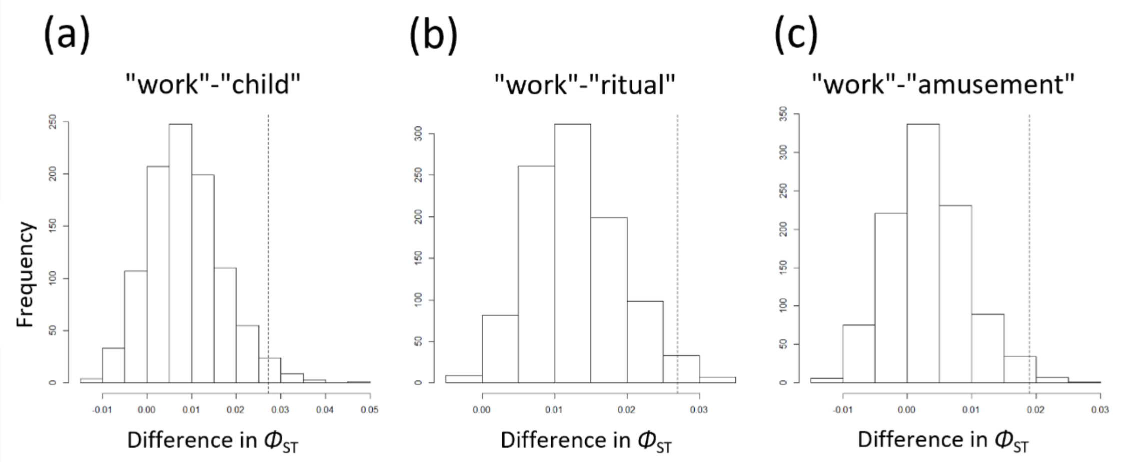

S2 Fig. Comparison of Φ_{ST} values between social contexts. Each panel shows the frequency distribution of the difference in the Φ_{ST} values for the five regions (Fig. 1) between different social contexts calculated from 1,000 bootstrap samples. The observed differences in Φ_{ST} are indicated by the vertical dashed lines. Φ_{ST} of "work" songs was significantly larger than that of (a) "child" songs ($p = 0.023$), (b) "ritual" songs ($p = 0.023$), and (c) "amusement" songs ($p = 0.012$).
